# Occurrence and abundance of zoonotic nematodes in snapper *Chrysophrys auratus*, a popular table fish from Australian and New Zealand waters

**DOI:** 10.1016/j.fawpar.2021.e00120

**Published:** 2021-03-16

**Authors:** Md. Shafaet Hossen, Skye Wassens, Shokoofeh Shamsi

**Affiliations:** a School of Animal and Veterinary Sciences & Graham Centre for Agricultural Innovation, Charles Sturt University, Wagga Wagga, NSW 2678, Australia; b Department of Fisheries Biology and Genetics, Bangladesh Agricultural University, Mymensingh 2202, Bangladesh; c School of Environmental Sciences & Institute of Land, Water and Society, Charles Sturt University, Albury, NSW 2640, Australia

**Keywords:** Anisakidae, Cucullanidae, Fish, Zoonotic nematodes, Seafood safety, Public health

## Abstract

In Australia and New Zealand (NZ), snapper *Chrysophrys auratus* is known for delicate mild flavoured flesh and is a favoured species to serve raw as sashimi or in sushi. The diet of snapper includes a variety of intermediate hosts of larval nematodes, and as a result, snapper has potential to become highly infected with zoonotic/non-zoonotic nematodes. The aims of this study were to survey nematodes in snapper from Australia and New Zealand waters and to identify nematode species using combined morphological and molecular methods. The zoonotic potential of nematodes identified in this study are discussed. A total of 112 snapper were purchased from the Sydney fish market, New South Wales, Australia. Fish were dissected and only the visceral content and digestive tract were examined for nematode infection. Parasites were initially identified by the microscopic method as four different types belonging to the families Anisakidae (*Anisakis* types I & III, and *Terranova* type II) and Cucullanidae (*Dichelyne* spp.). All Anisakidae nematodes were at infective stages. Species-level identification was actualised through sequencing of the internal transcribed spacer (ITS–1, 5.8S, ITS–2) regions. The *Anisakis* types I & III were confirmed as *Anisakis pegreffii* and *A. brevispiculata*, respectively of which *A. pegreffii* is considered globally as a zoonotic nematode. The specific identification of *Terranova* type II and *Dichelyne* spp. was not possible as no comparable sequence data were available in GenBank. The phylogenetic tree clustered *Anisakis* types I & III with *A. pegreffii* and *A. brevispiculata*, respectively; *Terranova* type II sequences as a separate clade with previously identified larval and adult *Terranova* and *Pseudoterranova* species. Based on phylogenetic analyses the present Cucullanid specimens were assigned herein as *Dichelyne* cf. *pleuronectidis*, and an unknown species *Dichelyne* sp. 1. This study represents the first host record globally for zoonotic Anisakid nematodes in this popularly consumed table fish and a new region record for *D.* cf. *pleuronectidis* and *Dichelyne* sp. 1. Further investigation is required, using more comprehensive parasite detection and recovery methods, to assess the health risk these nematodes may pose to human and fish health in Australia/NZ.

## Highlights

• First evidence of zoonotic *Anisakis pegreffii* infection in snapper *Chrysophrys auratus.*
• First evidence of *A. brevispiculata* and *Terranova* type II infection in snapper.
• Snapper was the verified host for *Dichelyne* cf. *pleuronectidis* and *Dichelyne* sp. 1.
• Infective stage of *A. pegreffii* was the highest prevalent nematode found in snapper.

## 1 Introduction

The snapper *Chrysophrys auratus* (Perciformes: Sparidae) is important to Australian recreational, commercial (Kailola et al., 1993; Norriss and Crisafulli, 2010), and charter industries. Snapper is also an important food species for indigenous Australians (FRDC, 2019) and is a revered fish within their spirituality and traditional knowledge (Prober et al., 2011). It is considered one of the nation's most important and iconic fish (Fowler et al., 2017; Norriss and Crisafulli, 2010) and is a popular and well-known table species that returns high prices at the retail level (FRDC, 2019). Australia/New Zealand (NZ), as multicultural nations, have embraced a range of imported cultural cuisines, including sushi and sashimi. According to the Kobe Jones Blog (Kobe Jones, 2019) snapper in Australia is considered suitable for sashimi and is regularly recommended by Australian chefs as compatible to use in raw fish dishes (Allen, 2019; Kwong, 2015).

Snapper is distributed in warm to temperate Indo-Pacific waters which occur off Australia/NZ (Paulin, 1990). Snapper is a long-lived predatory fish (Norriss and Crisafulli, 2010), and feed predominantly on crustaceans and small fish which are the intermediate hosts of many nematode species (Godfriaux, 1969; Gregori et al., 2015; Hurst, 1984). As a result, snapper have the potential to become highly parasitised. There have been no contemporary studies using combined morphological and molecular tools to accurately describe nematode parasites of snapper in Australian/NZ waters (Table 1). The most detailed parasitological examination of snapper nematodes was conducted by Johnston and Mawson (1945) and Sharples and Evans, 1995a, Sharples and Evans, 1995b, Sharples and Evans, 1995c in Australia and NZ waters, respectively. Both studies relied on morphological methods for nematode identification. However, few morphological markers of taxonomic significance for reliable species identification of larval nematodes are available (McManus and Bowles, 1996). During the last two decades, the application of molecular tools for the identification of nematodes has greatly advanced their taxonomy. Therefore, the primary aim of this study was to investigate the potential of snapper from the waters of Australia/NZ to be infected with nematode parasites. The secondary aim was to accurately identify these nematode parasites using combined morphological and molecular tools.

**Table 1:** Previous records of nematodes identified from the snapper *Chrysophrys auratus* in Australia and New Zealand.

Nematode	Microhabitat	Family	Location	Reference
*Anisakis* sp. larva	Encapsulated on viscera, mesenteries, and peritoneum of the body cavity	Anisakidae	Hauraki Gulf (Okakari Point and Kawau Bay), NZ	Sharples and Evans, 1995a, Sharples and Evans, 1995b
*Cucullanus* sp.	Intestine	Cucullanidae	Hauraki Gulf (Okakari Point and Kawau Bay), NZ	Sharples and Evans, 1995a, Sharples and Evans, 1995b
*Philometra lateolabracis*	Gonads	Philometridae	Hauraki Gulf (Okakari Point and Kawau Bay), NZ	Sharples and Evans, 1995a, Sharples and Evans, 1995b
*P. lateolabracis*	–	Philometridae	NZ	Hine and Anderson (1981)
*Anisakis* sp. larva	–	Anisakidae	NZ	Brunsdon (1956)
*Dichelyne cnidoglanis*	–	Cucullanidae	NZ	Brunsdon (1953)
*Hysterothylacium* sp.	–	Raphidascarididae	NZ	Brunsdon (1953)
*Dichelyne sheardi*	–	Cucullanidae	Outer harbour, SA	Johnston and Mawson (1949)
*Dichelyne sheardi*	–	Cucullanidae	Glenelg, SA	Johnston and Mawson (1945)
*Echinocephalus uncinatus* larva	Mesentery or omentum	Gnathostomatidae	Glenelg, SA	Johnston and Mawson (1945)
*Anisakis* larval type⁎	–	Anisakidae	Glenelg and Cape Jervis, SA	Johnston and Mawson (1945)

‘–’ indicates no information available; Abbreviations: NZ = New Zealand, SA = South Australia.

⁎ *Anisakis marina* (= *Capsularia marina*; *Stomachus marinus*) has been reported from various hosts across Australian coasts (Johnston and Mawson, 1944, Johnston and Mawson, 1945, Johnston and Mawson, 1949). In these reports “marina” mostly refers to larval stage of the nematode. Therefore, *A. marina* is not considered a valid taxon but regarded as *Anisakis* larval type. Both *Capsularia* and *Stomachus* have later been synonymised with *Anisakis* (Shamsi, 2014).

## 2 Materials and methods

### 2.1 Fish collection

A total of 112 fish were purchased from the Sydney fish market, New South Wales (NSW), Australia. The fish had been sourced from three separate localities: off the coast of NSW (*n* = 44; 11/10/2018), off the coast of NZ ((*n* = 20; 28/07/2018), and (*n* = 30; 16/09/2019)), and an unknown location (*n* = 20; 29/08/2018). Fish were transferred to the Parasitology Laboratory of Charles Sturt University, Wagga Wagga Campus in an insulated ice-filled box.

### 2.2 Parasite collection

All fish from each batch were examined on the day of arrival at the University. Fish were dissected and examined for the presence of nematodes according to the method described in Shamsi and Suthar (2016b) using both visual examination and incubation method to ensure maximum recovery of nematodes. Firstly, the surface of all inner organs was thoroughly inspected for the presence of nematodes under a dissecting microscope (Leica EZ4 Stereo Microscope, China). Encysted nematodes were removed and placed in sterile Eppendorf tubes containing 70% ethanol. The alimentary canal was then split from mouth to anus and other internal organs gently separated and placed in a petri dish containing a small amount of ambient temperature water before examining again under a dissecting microscope for the presence of parasites. The internal organs were placed in containers with water and after vigorous shaking were left to incubate overnight at room temperature. Splitting the digestive tract and separating internal organs allows trapped nematodes an opportunity to be released into the water and provides greater parasite recovery. All collected parasites were washed in ambient temperature physiological saline and preserved in 70% ethanol and stored at room temperature for further morphological and genetic study. Laboratory temperature was kept at a constant 25 °C.

### 2.3 Morphological examination

A small piece from the mid-body of each nematode was excised for molecular study and the rest of the body (head and tail) were cleared with lactophenol for morphological study. This study was conducted through a microscope (Upright Motorized Microscope ECLIPSE Ni-E, Nikon, Japan) fitted with a computer screen. Anisakid nematodes were initially identified to genus level using morphology and morphometry of anterior and posterior ends, oesophagus, ventriculus, the position of excretory pore and nerve ring (Murata et al., 2011; Shamsi and Suthar, 2016a). Cucullanid nematodes were morphologically identified based on the presence of anterior pseudobuccal capsule, spicules, number and organisation caudal papillae, precloacal ventral sucker, and tail according to instructions in Li et al. (2014); Moravec et al. (2019); Yamaguti (1935); Yamaguti (1941). The morphometric and meristic characteristics of systematic importance were measured directly with an eyepiece micrometre (BX-43 Olympus Microscope, Olympus Corporation, Japan). All measurements were recorded in both micrometres and millimetres as the mean, followed by the range in parentheses. A dash (−) indicates that measurements could not be made or were not available. All drawings were made to scale with the aid of a drawing tube of the compound microscope (BX-43 Olympus Microscope, Olympus Corporation, Japan). The prevalence, mean intensity, and mean abundance of nematodes were calculated according to Bush et al. (1997).

### 2.4 Sequencing

Genomic DNA from nematodes was extracted by DNeasy Blood & Tissue Kits (QIAGEN, Germany) and eluted by 40 μl of elution buffer. A volume of 25 μl PCR reaction was conducted to amplify the whole ITS (ITS–1, 5.8S, and ITS–2) regions of Anisakid nematodes using the primer sets of forward (SS1): 5′–GTTTCCGTAGGTGAACCTGCG–3′ and reverse (NC2): 5′–TTAGTTTCTTTTCCTCCGCT–3′. The cycling condition was followed according to Hossen and Shamsi (2021); Shamsi et al. (2020). A similar volume (25 μl) of PCR for the Cucullanid nematodes was conducted to amplify the whole ITS regions using another primer set, which included forward ITS–F: 5′–CCTAACAAGCCTCAACGGGTG–3′ and reverse ITS–R1: 5’–GCATACGAACTGAGAGCAGCG–3′ with the cyclic conditions of initial 95 °C for 2 min, then 95 °C for 30 s, 60 °C for 45 s, 72 °C for 1 min × 40 cycles following extension at 72 °C for 10 min and finally at 4 °C. An aliquot (3 μl) of each amplicon from both Anisakid and Cucullanid nematodes was examined on a 1.5% *w*/*v* agarose gel after staining with GelRed™ and photographed using a gel documentation system.

Representative samples were chosen from each group to send for sequencing to the Australian Genome Research Facility (AGRF). Identical primers set as for PCR were used to prepare the samples to send for sequencing. Sequence data including chromatogram were observed initially through Sequence Scanner software (Applied Biosystems® Genetic Analysers). Subsequently, sequences were aligned by MUSCLE (in MEGA v. 7) (Kumar et al., 2016) and then adjusted manually wherever necessary. Evolutionary analyses were conducted in MEGA v. 7 (Kumar et al., 2016).

### 2.5 Construction of phylogenetic tree

Two phylogenetic trees were constructed (based on two groups identified: Anisakids and Cucullanids) from the sequences generated in this study along with the representative sample sequences from GenBank (Table 2). GenBank sequences were sorted based on the BLAST searches and from the available publications. The phylogenetic relationships among specimens were calculated by the Bayesian method using MrBayes v 3.2 (Ronquist and Huelsenbeck, 2003). The GTR + G model was applied for both trees as suggested by jModelTest 2 (Darriba et al., 2012). *Dichelyne romani* (GenBank accession: KP699576) and *Hysterothylacium aduncum* (GenBank accession: KY909270) were used as outgroups for Anisakid and Cucullanid nematodes, respectively. For the construction of both phylogenetic trees, the sample frequencies were set at 1000, and calculated for 10,00,000 generation until the *p* value reached <0.01. After the mcmc run, the first 30% samples were discarded, and the sum command was used to summarise the phylogenetic trees. The phylogenetic tree was visualised using Figtree v 1.4.3 (Rambaut, 2014).

**Table 2:** Details of the specimens used to construct the phylogenetic trees.

Nematode specimen	GenBank accession number	Host scientific name	Host common name	Geographical origin of the specimen	Reference
*Anisakis pegreffii*	AY821740	*Lissodelphis borealis*	Northern right whale dolphin	Drakes Beach, California, USA	Nadler et al. (2005)
*A. simplex* S. S.	AY826723	*Trachurus trachurus*	Atlantic horse mackerel	Cantabrian Sea, Spain	Nadler et al. (2005)
*A. typica*	AY826724	*Stenella longirostris*	Spinner dolphin	Coast of Brazil	Nadler et al. (2005)
*A. physeteris*	AY826721	*Physeter catodon*	Sperm whale	Tyrrhenian Sea, Italy	Nadler et al. (2005)
*A. brevispiculata*	MK325199	*Kogia breviceps*	Pygmy sperm whale	Southeast of Melbourne, Australia	Shamsi et al. (2019b)
*A. berlandi*	MK325187	*K. breviceps*	Pygmy sperm whale	Southeast of Melbourne, Australia	Shamsi et al. (2019b)
*A. paggiae*	MK325218	*K. breviceps*	Pygmy sperm whale	Southeast of Melbourne, Australia	Shamsi et al. (2019b)
*A. nascettii*	JQ912692	*Mesoplodon grayi*	Gray's beaked whale	Off the NZ coast	Mattiucci et al. (2014)
*A. ziphidarum*	JQ912691	*Ziphius cavirostris*	Cuvier's beaked whale	Off the South African coast	Mattiucci et al. (2014)
*Contracaecum rudolphii* D	FM210251+FM210261	*Phalacrocorax carbo*	Great cormorant	NSW and Victoria, Australia	Shamsi et al. (2009a)
*C. rudolphii* E	FM210257+FM210269	*Phalacrocorax varius*	Pied cormorant	NSW and Victoria, Australia	Shamsi et al. (2009a)
*C. pyripapillatum*	AM940062+AM940066	*Pelecanus conspicillatus*	Australian pelican	Victoria, Australia	Shamsi et al. (2008)
*C. multipapillatum* D	AM940056+AM940060	*Pelecanus conspicillatus*	Australian pelican	Victoria, Australia	Shamsi et al. (2008)
*C. bancrofti*	EU839566+FM177880	*Pelecanus conspicillatus*	Australian pelican	Victoria, Northern Territory, NSW, Australia	Shamsi et al. (2009b)
*C. microcephalum*	FM177524+FM177528	*Phalacrocorax melanoleucos*	Little pied cormorant	Lara and Healesville, Victoria, Australia	Shamsi et al. (2009b)
*C. variegatum*	FM177531+FM177541	*Anhinga melanogaster* and *Pelecanus conspicillatus*	Australian darter and Australian pelican	Melbourne, Victoria.	Shamsi et al. (2009b)
*C. eudyptulae*	FM177550+FM177578	*Eudyptula minor*	Little penguin	Victoria, Australia	Shamsi et al. (2009b)
*C. ogmorhini*	FM177542+FM177549	*Arctocephalus pusillus doriferus* and *A. forsteri*	Australian and New Zealand fur seals	Victoria, Australia	Shamsi et al. (2009b)
*Mawsonascaris vulvolacinata*	MK476521	*Pastinachus ater*	Cowtail stingray	Queensland, Australia	Shamsi et al. (2019c)
*Terranova* type I	MT635348	*Platycephalus bassensis*	Sand flathead	NSW, Australia	Hossen et al. (2021)
*Pulchrascaris australis*	MK890747	*Sphyrna lewini*	Scalloped hammerhead shark	Off Cairns, Australia	Shamsi et al. (2020)
*Terranova* type II	MT635350	*Platycephalus richardsoni*	Tiger flathead	NSW, Australia	Hossen et al. (2021)
*T. pectinolabiata*	MK542878	*S. mokarran*	Great hammerhead shark	NSW, Australia	Shamsi et al. (2019a)
*Pseudoterranova azarasi*	AJ413973+AJ413974	*Eumetopias jubatus*	Steller sea lion	Iwani, Japan	Zhu et al. (2002)
*P. bulbosa*	AJ413970+AJ413971	*Erignathus barbatus*	Bearded seal	Newfoundland, Canada	Zhu et al. (2002)
*P. cattani*	AJ413982+AJ413984	*Otaria byronia*	South American sea lion	Concepcion, Chile	Zhu et al. (2002)
*P. decipiens*	AJ413967+AJ413968	*Phoca vitulina*	Harbour seal	Newfoundland, Canada	Zhu et al. (2002)
*P. krabbei*	AJ413965+AJ413980	*Halichoerus grypus*	Gray seal	Froya Island, Norway	Zhu et al. (2002)
*Raphidascaris acus*	AY603537	*Anguilla anguilla*	European eel	Vistula Lagoon, Poland	Kijewska et al. (2008)
*Dichelyne pleuronectidis*	KF470872–83	*Pleuronichthys cornutus*	Ridged-eye flounder	East China Sea	Li et al. (2014)
*D. romani*	KP699576	*Notacanthus chemnitzii*	Snub-nosed spiny eel	Northeast Atlantic	Isbert et al. (2015)
*D. szidati*	MK131263	*Acanthistius patachonicus*	Argentine sea bass	Argentina	Unpublished
*Hysterothylacium aduncum*	KY909270	*Peltorhamphus novaezeelandiae*	New Zealand sole	Off the coast of Otago, NZ	Anglade and Randhawa (2018)
*H. australe*	HE862216+HE862223	*Seriola lalandi*	Yellowtail amberjack	Port Augusta, South Australia	Shamsi (2016)
*H. brucei*	HE862222+HE862230	*Kajikia audax*	Striped marlin	Nelson Bay, NSW, Australia	Shamsi (2016)
*H. kajikiae*	HE862220+HE862226	*Kajikia audax*	Striped marlin	Nelson Bay, NSW, Australia	Shamsi (2016)
*Anisakis* type I	MT791088–103	*Chrysophrys auratus*	Snapper	Australia and NZ	Present study with voucher numbers 169, 177, 231–2, 232–1, 235, 236–1, 239, 246–1, 247, 252–1, 258–1, 285, 291, 420, 427, and 440
*Anisakis* type III	MT791104	*C. auratus*	Snapper	Australia	Present study with voucher number 413
*Terranova* type II	MT791105–06	*C. auratus*	Snapper	NZ	Present study with voucher numbers 168 and 252–5
*Dichelyne* cf. *pleuronectidis*	MT791107–10	*C. auratus*	Snapper	Australia and NZ	Present study with voucher numbers 91–4, 93–1, 281–1, and 283
*Dichelyne* sp. 1	MT791111	*C. auratus*	Snapper	Australia	Present study with voucher number 282

Abbreviations: NZ = New Zealand, NSW = New South Wales.

NB: Single GenBank accession indicates the whole ITS (ITS–1, 5.8S, ITS–2) sequence. Two ITS sequences connected with ‘+’ represent ITS-1 and ITS-2 sequences, respectively.

## 3 Results

### 3.1 Morphological identification of nematodes

**Fig. 1:**
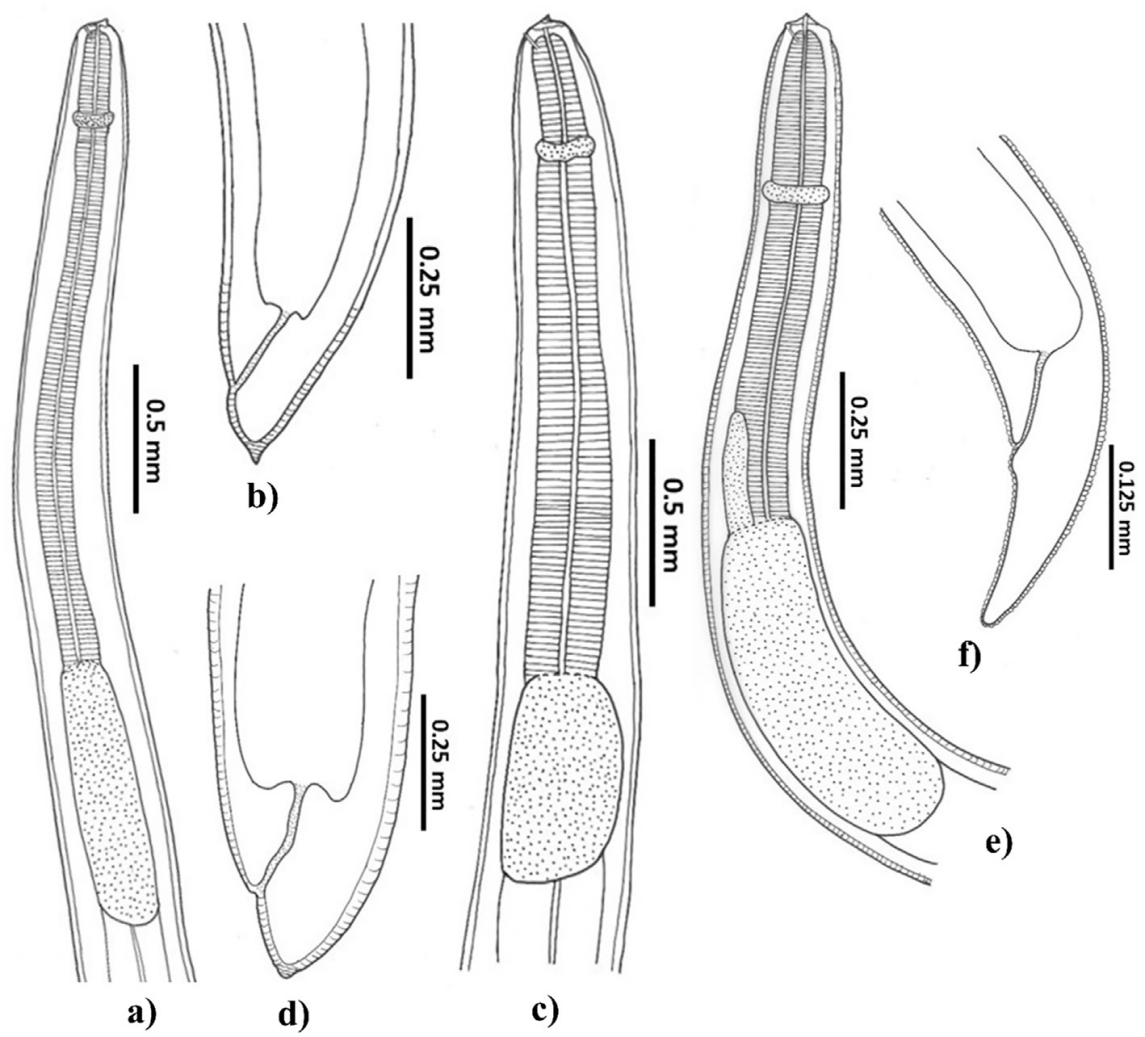
Morphology of Anisakid nematodes identified from snapper *Chrysophrys auratus*. a) Anterior end and b) Posterior end of *Anisakis* type I; c) Anterior end and d) Posterior end of *Anisakis* type III; e) Anterior end and f) Posterior end of *Terranova* type II.

**Fig. 2:**
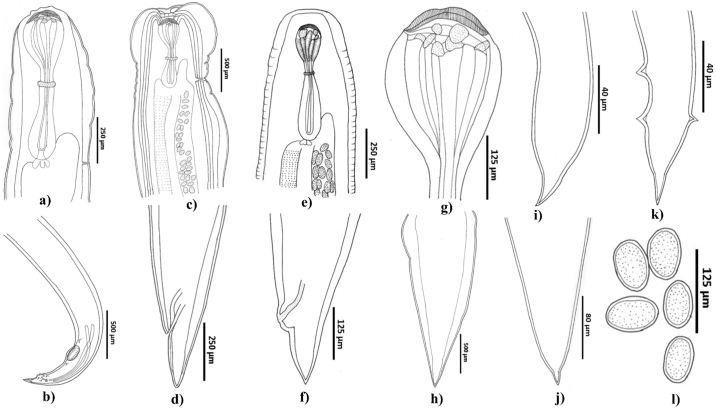
Morphology of Cucullanid nematodes identified as *Dichelyne* (*Cucullanellus*) spp. from snapper *Chrysophrys auratus*. a) Anterior end and b) Posterior end of mature male *Dichelyne* cf. *pleuronectidis*; c) Anterior end and d) Posterior end of gravid female *Dichelyne* cf. *pleuronectidis*; e) Anterior end and f) Posterior end of gravid female *Dichelyne* sp. 1; g) A typical pseudobuccal capsule; h–j) Posterior end of immature female specimens; k) Posterior end of immature male specimen; l) Eggs.

A total of four different nematode morphotypes belonging to the families Anisakidae and Cucullanidae were identified in this study. All Anisakid nematodes were identified as infective L3 larvae of *Anisakis* types I & III (Figs. 1a–d), and *Terranova* type II (Fig. 1e–f). Larvae were found in the digestive tracts, gonads, and liver. All Cucullanid nematodes were extracted only from the digestive system of the examined fish and were morphologically identified as *Dichelyne* (*Cucullanellus*) spp. which included larvae and adults (Figs. 2a–l). Among all nematode morphotypes identified in this study, *Anisakis* type I occurred in all samples/batch and had the highest prevalence of infection. The second most prevalent nematode morphotype was identified as *Dichelyne* spp. *Anisakis* type III and *Terranova* type II larvae were the least prevalent nematode morphotypes identified in this study. The general epidemiological data are represented in Table 3.

**Table 3:** Occurrence and abundance of nematodes in snapper *Chrysophrys auratus* examined in the present study.

Source of fish (number examined)	Nematode	Number of fish infected	Range in infected fish	Prevalence (%)	Total number of parasites found	Mean intensity	Mean abundance
SFM, NSW, Australia Date: 29-08-2018 (*n* = 20)	*Anisakis pegreffii*⁎	3	1–2	15	4	1.33 ± 0.59	0.20
	*Dichelyne* cf. *pleuronectidis*	4	1–1	20	4	1 ± 0.61	0.20
	*Dichelyne* sp. 1⁎	1	1–1	5	1	1 ± 0.62	0.05
Off the coast of NSW, Australia Date: 11-10-2018 (*n* = 44)	*Anisakis pegreffii*	3	1–1	7	3	1 ± 0.66	0.10
	*Anisakis brevispiculata*⁎	1	1–1	2	1	1 ± 0.67	0.03
	*Dichelyne* cf. *pleuronectidis*	4	1–4	9	8	2 ± 0.69	0.18
Off the coast of NZ Date: 28-07-2018 (*n* = 20)	*Anisakis pegreffii*	2	1–1	10	2	1 ± 0.75	0.10
	*D.* cf. *pleuronectidis*	3	1–1	15	3	1 ± 0.78	0.15
	*Terranova* type II⁎	1	1–1	5	1	1 ± 0.81	0.05
Off the coast of NZ Date: 16-09-2019 (*n* = 28)	*Anisakis pegreffii*	9	1–6	32	25	2.78 ± 0.86	0.89
	*D.* cf. *pleuronectidis*	3	1–1	11	3	1 ± 0.78	0.11
	*Terranova* type II	1	1–1	4	1	1 ± 0.82	0.04

Abbreviations: SFM = Sydney Fish Market, NSW = New South Wales, NZ = New Zealand.

⁎ Asterisk indicates the new host records of these nematodes.

### 3.2 Molecular identification

Representative samples from each morphotype were subjected to sequencing to confirm the specific identity of nematodes as detailed below.

Sixteen specimens belonging to *Anisakis* type I were subjected to sequencing. The length of ITS regions was 857 bp long and identical. Our sequences were 100% identical to those belonging to adult *A. pegreffii* (accession number AY821740) identified from the Northern right whale dolphin *Lissodelphis borealis* in the Drakes Beach, California, USA (Nadler et al., 2005). A single specimen belonging to *Anisakis* type III had the ITS sequence of 804 bp long and was 100% identical with the adult *A. brevispiculata* (accession number MK325199) reported from a pygmy sperm whale *Kogia breviceps* in the Southeast of Melbourne, Australia (Shamsi et al., 2019b).

Two specimens belonging to *Terranova* type II were subjected to sequencing. The ITS sequences of both specimens were 893 bp long and identical. There was no identical or highly similar sequence available in the GenBank.

Five specimens (voucher numbers 91–4, 93–1, 281–1, 283, and 282) belonging to *Dichelyne* spp. which included larva, mature males, and gravid females were subjected to sequencing. The ITS sequences of four specimens were 930 bp long and showed 0–0.30% nucleotide variability. A search in GenBank showed 99% similarity with *D. pleuronectidis* (accession numbers KF470872–KF470883) identified from ridged-eye flounder *Pleuronichthys cornutus* in the East China Sea (Li et al., 2014). The length of the ITS sequence for the other specimen (voucher number 282; a gravid female Cucullanid) was 813 bp long which had no closest similarity with the registered GenBank sequences. The pairwise comparison between the first four ITS sequences and the later, generated in the present study, revealed a substantial nucleotide variability at 0–24.10% and considered herein as interspecific genetic variation (Fig. 5).

### 3.3 Phylogenetic analyses of the nematodes

**Fig. 3:**
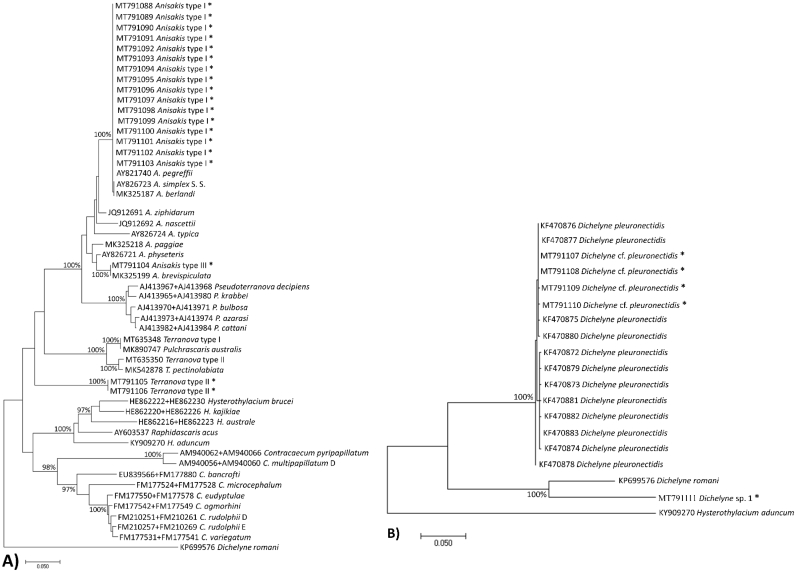
Phylogenetic relationship among the nematodes identified from snapper *Chrysophrys auratus* and those closely related species in GenBank (see Table 2 for details) inferred using Bayesian method with ITS (ITS-1, 5.8S, ITS-2) sequences. A) Phylogenetic tree for Anisakid nematodes; B) Phylogenetic tree for *Dichelyne* spp. nematodes. *indicates the ITS sequences generated in this study. Bayesian posterior probability values (%) were shown on the node.

The Bayesian inference phylogenetic tree clustered Anisakid nematodes found in this study with the members of Anisakidae identified previously in the literature (Fig. 3A). *Anisakis* type I independently grouped with members of *A. pegreffii* and *Anisakis* type III with *A. brevispiculata* demonstrating 100% posterior probabilities, respectively. *Terranova* type II larva found in the present study clustered into a separate clade and revealed a clear distinction with previously identified larval and adult *Terranova* and *Pseudoterranova* specimens in Australian waters and elsewhere with 100% posterior probability value. The Cucullanid nematodes found in the present study grouped with closely related GenBank *Dichelyne* specimens. Four sequences explored in this study clustered with the *D. pleuronectidis* with 100% posterior probability value (Fig. 3B). A single sequence obtained from a gravid female Cucullanid was isolated from the existing GenBank *Dichelyne* species with a 100% posterior probability value. The isolated specimen in this study was assigned herein as *Dichelyne* species 1 (Fig. 3B).

## 4 Discussion

This study confirmed the presence of *A. pegreffii*, *A. brevispiculata*, *Terranova* type II, *D.* cf. *pleuronectidis*, and *Dichelyne* sp. 1 infection in snapper from coastal waters of NSW and NZ. This is the first time that infectious stage larvae of the zoonotic *A. pegreffii* and potentially zoonotic *Terranova* type II have been identified in this species of snapper. New host records for *A*. *brevispiculata*, *D.* cf. *pleuronectidis*, and *Dichelyne* sp. were also established.

The individual prevalence of Anisakid and Cucullanid nematodes were 18% and 13%, respectively and for zoonotic *A. pegrefii*, the overall prevalence was 15%. This differs from Sharples and Evans (1995a) where *Anisakis* sp. was found to be rare with limited prevalence and low abundance. In the same study, *Cucullanus* sp. was reported moderately abundant in snapper. Differences between the present study and Sharples and Evans (1995a) may be due to the epidemiological profile of nematodes changing over time and differences in genera identified in the previous study. A further investigation with a greater sample size and conducted at different time points is required to clarify nematode infection trends in snapper.

In the present study, *Anisakis* larval types were morphologically identified as types I & III (Figs. 1a–d). In morphological identification, *Anisakis* type I could not be distinguished from larvae of *A. pegreffii*, *A. simplex* S. S., *A. berlandi*, and *A. typica* (Mattiucci et al., 2018; Murata et al., 2011). Similarly, *Anisakis* type III could be the larvae of *A. brevispiculata*, *A. physeteris*, and *A. paggiae* (Mattiucci et al., 2018; Murata et al., 2011). Previously, *Anisakis* sp. larva was found from the same host in Australia (Johnston and Mawson, 1945) and NZ (Sharples and Evans, 1995b, Sharples and Evans, 1995c). However, specific identification of the larva was uncertain at the time due to a lack of molecular techniques. The phylogenetic tree showed a clear distinction between *Anisakis* types I & III found in this study and grouped them with previously identified adult *A. pegreffii* and *A. brevispiculata*, respectively (Fig. 3A). Therefore, the present study confirms snapper as a host of *A. pegreffii* and *A. brevispiculata.*

**Fig. 4:**
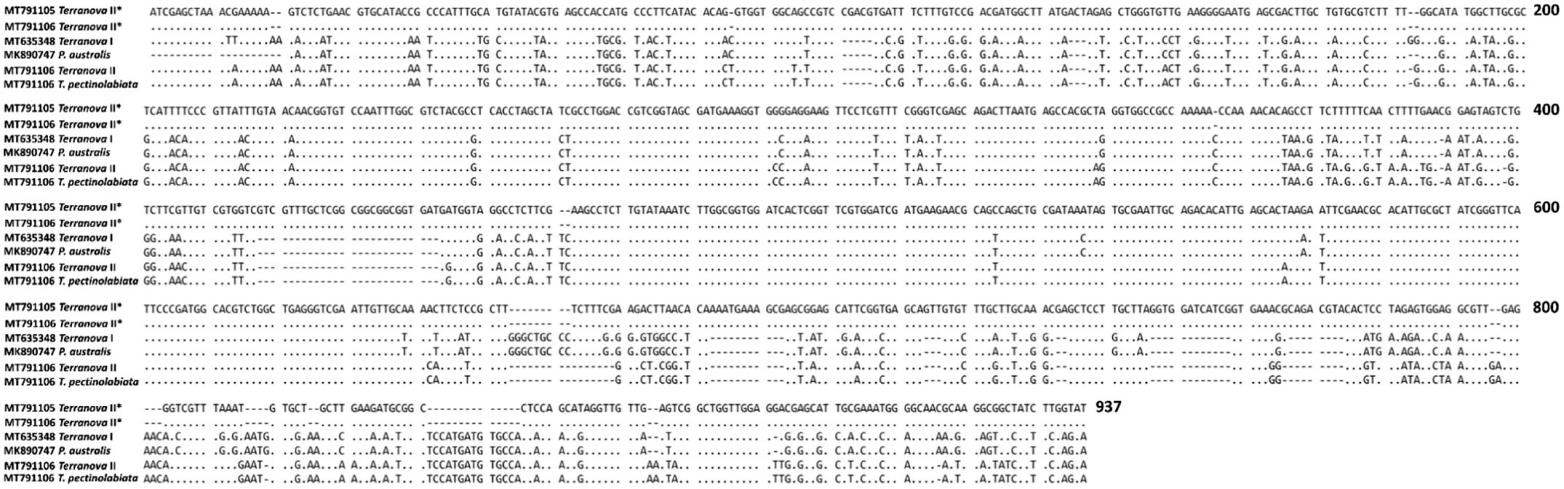
The ITS (ITS-1, 5.8S, ITS-2) sequence alignments of the present *Terranova* specimens and closely related species from GenBank. Sample's information is provided in Table 2. The dots represent identical bases and dashes indicate alignment gaps. The numbers at the right of alignments indicate the alignment position.

Another Anisakid nematode morphologically identified in this study was *Terranova* type II. *Terranova* types I and II have been previously identified and reported from other marine fish in Australia and New Caledonia (Hossen et al., 2021; Jabbar et al., 2012; Shamsi et al., 2018a; Hossen and Shamsi, 2019; Shamsi et al., 2018b; Shamsi and Suthar, 2016a) as *Pulchrascaris australis* and *T. pectinolabiata*, respectively (Shamsi et al., 2019a; Shamsi et al., 2020). Although, the morphological and morphometric data of the present specimens either partially or completely matched with previously identified *Terranova* larval types (Table 4) the molecular data did not match with existing sequences registered in GenBank and revealed 20.70–20.90% nucleotide variability (Fig. 4).

**Table 4:** Comparative measurements of *Terranova* larval types, for specimens found in the present study and previous studies.

	Present study	Jabbar et al. (2012)		Shamsi et al. (2018a)		Shamsi et al. (2016a)
Larval type	*Terranova* type II	*Terranova* type I	*Terranova* type II	*Terranova* type I	*Terranova* type II	*Terranova* type II
Locality	Off the coast of NZ	Lizard Island in the Great Barrier Reef, QLD, Australia		Province Sud, New Caledonia		Off Australian coasts including NSW, VIC, QLD, SA, and WA
Number of specimens observed	02	10	10	08	10	10
Body length	7.09 (6.8–7.38)	–	–	9.92 (7.5–12.55)	6.63 (5.42–8.30)	6.60 (3.00–9.00)
Maximum body width	0.24 (0.22–0.25)	–	–	0.25 (0.19–0.32)	0.23 (0.18–0.28)	0.24 (0.18–0.28)
Oesophagus length	0.92 (0.90–0.93)	1.16 (1.08–1.65)	0.94 (0.78–1.10)	1.00 (0.7–1.28)	0.85 (0.73–1.03)	0.88 (0.40–1.14)
Ratio of oesophagus length to body length (%)	12.98 (12.60–13.24)	–	–	10.08 (7.06–12.90)	12.82 (11.01–15.54)	14.30 (9.50–26.50)
Intestinal caecum length	0.88 (0.85–0.90)	1.52 (0.73–2.08)	0.72 (0.60–0.87)	1.23 (0.60–1.70)	0.68 (0.61–0.85)	0.71 (0.50–0.90)
Ventriculus length	0.57 (0.51–0.62)	1.32 (0.95–1.96)	0.38 (0.35–0.45)	1.09 (0.35–1.45)	0.34 (0.29–0.38)	0.38 (0.24–0.54)
Nerve ring to anterior end	0.29 (0.28–0.30)	0.30 (0.25–0.33)	0.28 (0.26–0.36)	0.27 (0.2–0.33)	0.25 (0.22–0.32)	0.37 (0.22–0.72)
Tail length	0.19 (0.18–0.20)	0.18 (0.16–0.20)	0.15 (0.12–0.18)	0.13 (0.11–0.17	0.13 (0.09–0.15)	0.13 (0.12–0.14)

All measurements are given in millimetres; mean followed by range in parentheses. ‘–’ indicates no measurements/data available; Abbreviations: NSW=New South Wales, VIC=Victoria, SA = South Australia, WA = Western Australia, QLD = Queensland, NZ = New Zealand.

Very little is known about *Terranova* larval types compared to other Anisakid nematodes (Moravec and Justine, 2020). The larvae belonging to the genera *Pulchrascaris*, *Terranova*, and *Pseudoterranova* look morphologically similar and are all referred to as *Terranova* larval types (Shamsi and Suthar, 2016a). The species belonging to the later genus are considered to be the second most common zoonotic nematode causing human illness after *Anisakis* spp. (Arizono et al., 2011; McClelland, 2002; Menghi et al., 2020; Torres et al., 2007; Weitzel et al., 2015). Although, *Terranova* type A larva has been reported for two cases of human infection in Korea (Lee et al., 1985; Seo et al., 1984), however this requires further investigation (Moravec and Justine, 2020). The phylogenetic tree grouped *Terranova* type II sequences obtained in the present study independently from the registered sequences for Ascaridoid nematodes larvae and adults in GenBank with 100% posterior probability value (Fig. 3A). The new sequences did not cluster with earlier larval and adult *Terranova* and *Pseudoterranova* species identified in the Australian waters and abroad. Therefore, the specific identification of the present *Terranova* type II requires clarification. Further parasitological examination with greater sample size is required to explore more genetic information for the specific identification of *Terranova* larval infection in snapper.

Although other nematodes (*Dichelyne* spp.) found in this study are not of zoonotic significance, they impact fish health and can significantly damage the intestinal wall and disrupt functional nutrient absorption leading to deficits in host growth, health and death (Dunn et al., 1983; Li et al., 2014; Rezaei et al., 2013). Until now, two *Dichelyne* (*Cucullanellus*) spp. have been reported from the Australian waters, *D. sheardi* from the silver spot *Chironemus maculosus* and snapper *C. auratus* as well as *D. cnidoglanis* from the estuarine catfish *Cnidoglanis macrocephalis* (Johnston and Mawson, 1944, Johnston and Mawson, 1945, Johnston and Mawson, 1949). In NZ, a single species *D. cnidoglanis* has been reported (Brunsdon, 1953; ex. from Sharples and Evans in 1995c) with reports of unidentified species as *Cucullanus* (not *Dichelyne* species) from snapper (Sharples and Evans, 1995c). The morphometric and meristic data suggest our specimens are different from those previously described from the Australian and NZ waters.

**Fig. 5:**
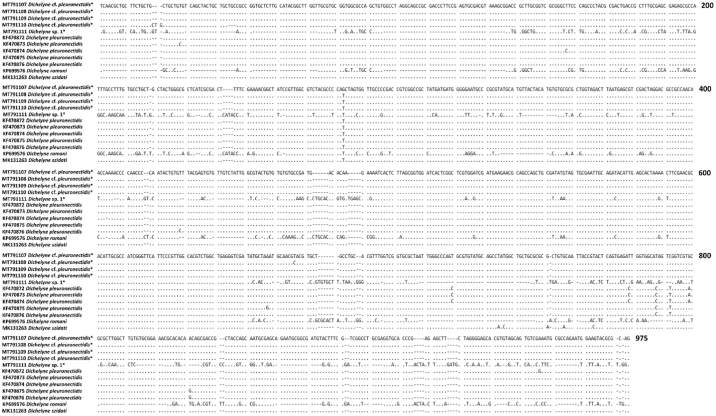
The ITS (ITS-1, 5.8S, ITS-2) sequence alignments of the present *Dichelyne* specimens and closely related species from GenBank. Sample's information is provided in Table 2. The dots represent identical bases and dashes indicate alignment gaps. The numbers at the right of alignments indicate the alignment position.

Globally, a total of three *Dichelyne* (*Cucullanellus*) spp. have been identified from the Sparid hosts (Isbert et al. (2015), *D. adriaticus* (Petter, 1974); *D. jialaris* (Moravec et al., 2018); and *D. pleuronectidis* (Li et al., 2014; Yamaguti, 1935; Yamaguti, 1941) of which our specimens most closely resembled *D. pleuronectidis* (Table 5). However, specimens in our study were different from previous descriptions in Li et al. (2014); Yamaguti (1935) and Yamaguti (1941) with respect to: i) markedly thick cuticle with transverse striations; ii) muscular oesophagus expanded at the anterior end to form a distinct pseudobuccal capsule; iii) anterior oesophageal region being much-expanded/wider than the posterior region and clearly distinguishable. The pairwise genetic comparison between the four ITS sequences generated in this study and those available in GenBank demonstrated 0–0.60% nucleotide variability (Fig. 5) with *D. pleuronectidis* thus the *Dichelyne* nematode identified in this study was assigned as *D.* cf. *pleuronectidis*. Further investigation is required to reach a solid conclusion if the observable morphological variations in *D.* cf. *pleuronectidis* found in the present specimens are due to geographical differences of host origin. In the present study, a single gravid female *Dichelyne* species herein named as *Dichelyne* sp. 1 remains unidentified due to the lack of comparable mature male specimens and revealed 0.10–24.40% nucleotide variability with those sequences registered in GenBank (Fig. 5). The phylogenetic tree clustered four (out of five) *Dichelyne* nematode sequences obtained in the present study with the sequences registered for *D. pleuronectidis* in GenBank. A single sequence from the present study grouped in isolation with 100% posterior probability value. The isolated specimen was herein named as *Dichelyne* sp. 1 (Fig. 3B). Further genetic analyses are required, for the morphologically identified *Dichelyne* nematodes, to verify their specific position.

**Table 5:** Comparative measurements of *Dichelyne* cf. *pleuronectidis* and *Dichelyne* sp. 1 from snapper *Chrysophrys auratus*, for specimens collected in the present study and previous studies.

	Present study				Present study	Li et al. (2014)		⁎Yamaguti (1935)		⁎Yamaguti (1941)	
Parasite	*Dichelyne* cf. *pleuronectidis*				*Dichelyne* sp. 1	*Dichelyne pleuronectidis*		*Dichelyne pleuronectidis*		*Dichelyne pleuronectidis*	
Host	Snapper *Chrysophrys auratus*				Snapper *C. auratus*	Ridged-eye flounder *Pleuronichthys cornutus*		Three Pleuronectids: *Pleuronichthys cornutus*, *Paralichthys olivaceus*, *Pseudorhombus cinnarnoneus*		Snapper *Pagrosomus unicolor* (Syn. *C. auratus*)	
Locality	Australia: SFM				Australia: SFM	China: The East China Sea		Japan: Toyama Bay; the Inland Sea; and Mutu Bay		Japan: The Inland Sea	
	New Zealand: Off the coast of NZ										
Specimen type (number measured)	Mature male (*n* = 3)	Immature male (*n* = 3)	Gravid female (*n* = 2)	Immature female (*n* = 5)	Gravid female (*n* = 1)	Mature male (*n* = 15)	Gravid female (*n* = 15)	Male (*n* = not specified)	Female (*n* = not specified)	Male (*n* = not specified)	Female (*n* = not specified)
Body length (mm)	4.76 (4.13–5.63)	3.14 (3.00–3.38)	7.22 (6.63–7.80)	5.56 (2.8–7.43)	4.03	6.67 (5.49–7.94)	7.40 (5.80–8.70)	3.15–8.00	5.50–11.00	3.70–4.40	4.00–11.00
Maximum body width	460 (380–500)	207 (180–250)	985 (670–1300)	556 (220–900)	450	328 (225–421)	391 (294–471)	175–350	300–650	270–320	260–700
Oesophagus length	800 (700–850)	523 (450–640)	975 (850–1100)	776 (500–950)	520	809 (735–882)	915 (833–980)	500–910	800–1120	600–700	740–1000
Maximum oesophagus width	173 (150–200)	100 (80–140)	210 (200−220)	184 (120−220)	100	170 (147–196)	–	75–250	125–270	120–130	110–240
Minimum oesophagus width	73 (60–80)	55 (40–75)	90 (80–100)	76 (40–90)	40	107 (88–137)	–	–	–	–	–
Ratio of oesophagus length to body length (%)	17 (15–21)	17 (15–19)	13 (13–14)	14 (12–18)	13	12 (10–15)	13 (11–15)	–	–	–	–
Pseudobuccal capsule length	290 (240–350)	215 (180–275)	365 (330–400)	310 (190–400)	200	70 (49–88)	64 (49–78)	–	–	–	–
Pseudobuccal capsule width	217 (160–260)	157 (120−200)	270 (240–300)	232 (190–270)	130	83 (69–108)	97 (78–118)	110–220	150–200	135–155	150–240
Intestinal caecum length	250 (200−300)	180 (150–180)	350 (320–380)	153 (80–200)	130	239 (98–394)	262 (69–415)	180–450	110–530	320–380	200–500
Intestinal caecum width	100 (80–100)	50 (40–50)	130 (110–150)	123 (40–200)	50	58 (39–69)	58 (49–69)	–	–	–	–
Excretory pore to anterior end	480 (430–530)	–	700 (590–810)	723 (670–820)	–	529 (501–588)	646 (508–784)	550–1450	800–1550	600	
Nerve ring to anterior end	433 (400–500)	253 (230−300)	495 (470–520)	456 (250–580)	300	347 (314–392)	380 (323–392)	200–400	280–500	260–300	300–430
Deirids to anterior end	650 (520–750)	410 (330–450)	900 (850–950)	845 (500–1050)	–	750 (559–902)	783 (539–902)	–	–	–	–
Ventral precloacal sucker length	215 (200−230)	–	–	–	–	–	–	–	–	–	–
Ventral precloacal sucker width	130 (110–150)	–	–	–	–	–	–	–	–	–	–
Ventral precloacal sucker to cloaca	377 (350–400)	–	–	–	–	487 (412–539)	–	510–650	–	–	–
Ventral precloacal sucker to posterior end	570 (530–600)	–	–	–	–	–	–	–	–	–	–
Spicule length	933 (900–1000)	–	–	–	–	975 (735–1176)	–	630–1030		890–930	
Ratio of spicule length to total body length (%)	20 (17–24)	–	–	–	–	15 (12–17)	–	–	–	–	–
Gubernaculum length	40 (40–45)	–		–	–	44 (40–54)	–	33–48	–	–	–
Number of precloacal papillae (pairs)	3	–	–	–	–	3	–	–	–	–	–
Number of paracloacal papillae (pairs)	4	–	–	–	–	4	–	–	–	–	–
Number of postcloacal papillae (pairs)	3	–	–	–	–	3	–	–	–	–	–
Total number of caudal papillae (pairs)	10	–	–	–	–	10	–	11	–	–	–
Tail length	173 (150–200)	165 (150–180)	270 (250–290)	310 (210–460)	130	186 (157–206)	268 (225–323)	130–180	200–280	150–160	200–250
Phasmids to posterior end	233 (200–250)	–	400 (350–450)	423 (370–450)	–	–	127 (118–147)	–	–	–	–
Eggs count	–	–	~100–400	–	~100	–	45	–	–	–	–
Eggs length	–	–	80 (70–80)	–	70	–	62 (59–69)	–	63–84	–	66–84
Eggs width	–	–	40 (40–50)	–	40	–	46 (39–49)	–	39–46	–	39–45

All measurements are given in micrometres unless otherwise stated; mean followed by range in parentheses. ‘–’ indicates no measurement/data available; Abbreviations: SFM = Sydney Fish Market, NZ = New Zealand.

⁎ Some of the measurements have been converted into micrometres.

The zoonotic potential of all *Anisakis* spp. are not yet clear (Aibinu et al., 2019; Mattiucci et al., 2018). In most cases nematode larvae infecting humans are often damaged on removal (Mattiucci et al., 2013) are identified morphologically or based on the assumption (Mattiucci and D'Amelio, 2014). Given that a range of genera within the family can frequently infect humans, it is likely that all members of the *Anisakis* genus are potentially zoonotic. Therefore, further research on the specific identification of these larvae in human cases of infection is required.

According to Bao et al. (2017a) anisakiasis (human infection caused by *Anisakis* nematodes) is an emerging zoonosis which is underestimated globally. These zoonotic nematodes are medically important and cause intestinal (Kojima et al., 2013), and less commonly extra-intestinal anisakiasis as well as allergic reactions (Mattiucci et al., 2017). *Anisakis pegreffii* has increasingly been identified in human cases of allergic (Mattiucci et al., 2013), intestinal (Moschella et al., 2004), and extra-intestinal anisakiasis (Guardone et al., 2018; Bao et al., 2019).

Identification of zoonotic and/or potentially zoonotic larvae from a popular Australian/NZ table fish such as the iconic snapper, considered a suitable species for consuming raw, is of concern for human health (Shamsi, 2020). According to Shamsi and Sheorey (2018), anisakidosis (human infection caused by the larval Anisakid nematodes) in Australia is of emerging importance and may correspond with the adoption of novel cuisines including the popular sushi and sashimi. Very recently, the first human anisakiasis, caused by *Anisakis* spp. larvae was diagnosed in NZ after consumption of a sushi rolls (Beig et al., 2019).

All anisakid nematodes found in this study were viable infectious third-stage larvae and may cause human illness if accidentally consumed in raw or partially cooked fish (Bao et al., 2017a; Bao et al., 2018; Buchmann and Mehrdana, 2016; Cipriani et al., 2016; Caldeira et al., 2021; D'Amico et al., 2014). *Anisakis pegreffii* as the dominant species in the present study and recent reports of other infected fish in the Asia Pacific region may support a southward shift in parasite distribution (Palm et al., 2017; Chen et al., 2018; Zhang et al., 2018). *Anisakis pegreffi* has been identified as the dominant species in pelagic blue mackerel *Scomber australasicus* (Taiwanese waters) (Chen and Shih, 2015), pelagic scombrid bullet tuna *Auxis rochei* (Indonesian waters) (Palm et al., 2017), white spotted *Conger Conger myriaster* (South China Sea) (Chen et al., 2018) and the yellow goosefish *Lophius litulon* (East China Sea) (Zhang et al., 2018). Further monitoring of fish in Australian waters, therefore, seems warranted.

Further research is required to determine the human health risks using a greater sample size covering both a greater temporal and spatial range. In addition, more advanced methods of parasite isolation should be considered in future studies as examining only the fish viscera in the present study limited the strength of the results. Candling of fish fillets is recommended in Codex Alimentarius ‘*Code of practice for fish and fishery products*’ for identification of parasites (Codex Alimentarius, 2020) in fish musculature. However, this method is less effective in detection of *Anisakis* spp. and darker *Pseudoterranova* spp. larvae (Levsen et al., 2005; McGladdery, 1986; Petrie et al., 2007; Mercken et al., 2020a). Candling combined with pressing may be more effective to detect nematode parasites in the musculature of fish (Karl and Leinemann, 1993). Levsen et al. (2005) found that in blue whiting with an average thickness of 11 mm, the detection of nematodes using UV light was only 10–15% however Gómez-Morales et al. (2018) found the UV press method had a high sensitivity for detection of nematodes in fish musculature and viscera. Artificial pepsin digestion of fish musculature is also recommended in the Codex fishery code of practice for recovery of parasites in high-risk fish species (Codex Alimentarius, 2020) and fillets (Mercken et al., 2020b). This results in the total destruction of the fish and for commercial purposes is impractical. However, with the optimisation of this technique described in Llarena-Reino et al. (2013) the pepsin digestion method in the experimental setting would be an effective, low-cost, and accessible alternative to advanced methods such as Magnetic Resonance Imaging (MRI) described in Bao et al. (2017b). Press method candling in combination with artificial digestion of fish fillets in future studies will provide a more accurate indication of the potential of snapper fillets to be infected with zoonotic nematodes larvae.

Also, of importance is the potential for cross-contamination as a human health concern. *Anisakis simplex* has been identified as an important hidden allergen in food (Anibarro et al., 2007) and Bao et al. (2019) considers all *Anisakis* spp. as potential and important food allergens. As *A. pegreffii* has been identified as the causative agent in cases of human allergic anisakiasis the potential for cross-contamination of viable larvae from viscera onto fillets should be considered as a human health concern.

Seafood borne parasitic disease in Australia/NZ is little recognised or acknowledged. *The Australia New Zealand Food Standards Code* (ANZFSC, 1997) has not included information on fish borne parasites in local fish. The *‘The Compendium of Microbiological Criteria for Food (2018)’ from Food Standards Australia and NZ* in Appendix I (FSANZ, 2018) mentioned parasites as a possible pathogenic microorganism that can cause foodborne illness only once.

In conclusion, snapper sourced from the waters of Australia and NZ was identified infected with zoonotic, potentially zoonotic, and non-zoonotic nematodes. Further research into zoonotic nematodes in snapper using advanced detection methods will identify if current Australian food safety regulations regarding zoonotic or potentially zoonotic nematodes in fish and fishery products in Australia/NZ require updating. Fish is an excellent source of lean protein and contains many beneficial fatty acids essential for early development as well as eye, brain and cardiovascular health (Aadland et al., 2015). The identification of zoonotic and potentially zoonotic parasites should not be a deterrent to regular consumption of this healthy protein. If intended for consumption raw or lightly processed, it is recommended that fish be frozen at ≤ − 20 °C for a minimum period of one day (EC, 2004). Adequately cooking, where the internal temperature of fish reaches ≥55 °C, for a minimum period of 5 min will largely negate the risks to human health (De Marval et al., 2013).

## Animal ethics

Not applicable.

## Declaration of Competing Interest

Md. Shafaet Hossen, Sky Wassens, and Shokoofeh Shamsi declare that they have no conflict of interest.
